# Overexpression of Endometrial Estrogen Receptor-Alpha in The Window of Implantation in Women with Unexplained Infertility

**DOI:** 10.22074/ijfs.2018.5118

**Published:** 2018-01-15

**Authors:** Mehran Dorostghoal, Hamid-o-allah Ghaffari, Farideh Moramezi, Narjes Keikhah

**Affiliations:** 1 Department of Biology, Faculty of Science, Shahid Chamran University of Ahvaz, Ahvaz, Iran; 2 Hematology-Oncology and Stem Cell Transplantation Research Center, Tehran University of Medical Science, Tehran, Iran; 3 Fertility, Infertility and Perinatology Research Center, Ahvaz Jundishapur University of Medical Science, Ahvaz, Iran

**Keywords:** Estrogen Receptor-α, Glycodelin-A, Implantation

## Abstract

**Background:**

Failure in the endometrial receptivity may account for a significant number of infertility cases including unexplained infertility in women. Reduction in the endometrial estrogen receptor-alpha (*ER-α*) expression during implantation may be a critical event that coincides with the expression of specific genes and the formation of a receptive endometrium. The aim of the present study was to assess the expression of *ER-α* in the mid-secretory phase in the endometrium of women with unexplained infertility.

**Materials and Methods:**

This case-control study was carried out on randomly selected fertile (n=10) and infertile (n=16) women whose source of infertility remained unexplained. We evaluated the expression of *ER-α* and glycode-lin-A (*GdA*) through mRNA level measurement with real-time polymerase chain reaction (PCR) in the endometrium of fertile women and patients suffering from unexplained infertility and fertile women. Endometrial biopsies of each subject were collected during a single menstrual cycle 7 days after the peak of luteinizing hormone (LH+7).

**Results:**

Endometrial expression level of *ER-α* was significantly (P<0.05) higher in the patients with unexplained infertility compared to the control. Significantly (P<0.05) lower levels of *GdA* expression were seen in women with unexplained infertility. A statistically non-significant negative correlation was observed between *ER-α* and *GdA* mRNA expression.

**Conclusion:**

Our findings demonstrate that reduction in the endometrial *GdA* expression is associated with elevated expression of *ER-α* in mid-luteal phase. Disruption in the endometrial *ER-α* expression, which leads to defects in uterine receptivity, may contribute to unexplained infertility.

## Introduction

Endometrial receptivity plays a key role in the establishment of a successful implantation and its impairment may contribute to infertility in women (1). A variety of molecules such as hormones, receptors, adhesion molecules, growth factors and cytokines mediate the embryomaternal crosstalk and facilitate the reception of a blastocyst and the establishment of implantation (2). During the menstrual cycle uterine receptivity is regulated by the secretion of the ovarian steroids. Endometrial proliferation is induced by estrogen during the preovulatory phase, whereas progesterone causes secretory changes in the estrogen- primed endometrium (3).

Ligand-specific intracellular receptors located in stro. mal and epithelial endometrial cells mediate the actions of estrogen and progesterone (4). It is thought that the pres. ence of progesterone after appropriate estrogen priming is required to stimulate key implantation-specific events in the mid-secretory phase of the menstrual cycle (5).

Estrogen receptor-alpha (*ER-α*) increases during the proliferative phase in response to estrogen and is downregulated during the window of implantation in response to progesterone (6). The disappearance of *ER-α* at the time of implantation has been reported in most mammalian species (7). The decline in *ER-α* coincides with endometrial gene expression in the mid-luteal phase, and is a critical event in the establishment of endometrial receptivity (8). High levels of *ER-α* during implantation were observed in women with polycystic ovarian syndrome (PCOS) and endometriosis. Elevated expression of *ER-α* in both groups of patients was associated with the reduction in beta 3 integrin expression, a marker of endometrial receptivity (9). It has been suggested that the disappearance of *ER-α* at the time of implantation may disturb the expression pattern of proteins that regulate the endometrial receptivity.

Glycodelin-A (*GdA*) is a progesterone-regulated glycoprotein with immunosuppressive properties that is highly upregulated in glandular epithelium at implantation and plays a role in the formation a receptive endometrium (10). *GdA* expression is concurrent with pinopode formation in the receptive endometrium (11), indicating that it can potentially be seen as a diagnostic marker of morphological differentiation of human endometrium (12). A lower glycodelin expression in secretory phase was found in eutopic endometrium of endometriosis patients and in uterine flushings from women with unexplained infertility when compared to the healthy controls (13, 14). 

Assuming that unexplained infertility can be due to disturbances in the molecular and the cellular biomarkers involved in implantation (15), we hypothesized that continued *ER-α* expression may be detrimental to the development of endometrial receptivity. In present study expression of *GdA*, as a particular marker of endometrial receptivity, was assessed at the time of implantation. 

## Materials and Methods

This case-control study was approved by the Research Ethics Committee of Shahid Chamran University of Ahvaz, Iran. The study was performed in the Laboratory of Embryology, Department of Biology. Written informed consent was obtained from each participant.

## Sample collection

Endometrial biopsy samples were collected using a Novak curette in the mid-luteal phase at day luteinizing hormone (LH)+7 from healthy volunteers women with proven fertility (n=10, age 32.5 ± 3.2 Y) and women with unexplained infertility (n=16, age 31.6 ± 3.0 Y) that showed primary infertility for more than 2 years (30.5 ± 4.7 months). The unfertile females were randomly selected from a population of such females listed in Imam Khomeini hospital medical records. Endometrial samples were divided into two parts. One sample was fixed in 10% formalin and embedded in paraffin. After tissue processing, 5-6 µm sections were stained with haematoxylin- eosin, evaluated histologically to correspond all samples to the assumed time in the cycle according to the Noyes et al. (16) criteria. The other sample was immediately stored in RNA later at -80°C for later use in real-time polymerase chain reaction (RT-PCR). Sample size was determined based on previous studies (17, 18). Sample size was smaller in the fertile group due to the low collaboration. The concentration of LH in morning urine (ACON Laboratories, Inc., USA) was used to determine the day of the surge.

All women included in this study had normal ovarian function and regular menstrual cycles, confirmed based on their menstrual histories, and none of them had used steroid hormones, (for at least 6 months prior to study), and intra-uterine contraceptives. Women with unexplained infertility showed normal ovulatory cycles and mid-luteal serum progesterone levels, normal tubal patency and no recognizable endometriosis based on symptoms and clinical examination in transvaginal ultrasonography or diagnostic laparoscopy. Moreover, unexplained infertile women had partners with normal semen according to WHO criteria. Patients with history of pelvic inflammatory diseases, pelvic surgery including cesarean section, unilateral tubal patency, ovarian hyperstimulation syndrome, diminished ovarian response, endometriosis or multiple female factor were excluded from this study.

## Hormone assay

Blood samples were obtained in the fasted state on the same day as endometrial sampling and serum levels of LH, follicle stimulating hormone (FSH), estradiol (E2), and progesterone (P4) were measured using commercially available kits (Abcam plc, UK).

## RNA extraction

Total RNA was extracted from the endometrial tissues (approximately 50-100 mg) using Tripure (Roche Diagnostics, Germany), according to the recommended protocol by the manufacturer. RNA integrity was analyzed via electrophoresis and total RNA concentration was obtained using a spectrophotometer at an optical density of 260 nm. The RNA was stored at -70°C for future procedures.

## cDNA synthesis

Synthesis of cDNA was carried out using 1 mg of total RNA from each sample with random hexamer primers using prime Script™ RT reagent Kit (Takara Bio Inc., Japan) according to the manufacturer’s instructions.

## Quantitative real-time polymerase chain reaction analysis

Real-time PCR was performed for relative quantification of the *ER-α* and *GdA* genes expression using ABI StepOne plus™ System (Applied Biosystems, Germany). Hypoxanthine phosphoribosyltransferase (HPRT) gene was used as the housekeeping gene. Forward and reverse primer sequences for each gene are presented in Table 1. The specificity of primers for each gene was analyzed in the BLAST database. The reaction mixture consisted of 10 µl Master mix SYBR Green, 2 µl cDNA, 1 µl of each primer (10 pmol/µl), and 7 µl dH_2_O (Qiagen, Germany). The standard cycling protocol used for all genes consisted of DNA denaturation and enzyme activation at 95°C for 10 minutes, denaturation 95°C for 15 seconds, annealing at 62°C for 15 seconds and extension and florescence acquiring at 72°C for 15 seconds. The RT-PCR procedure was carried out 40 cycles. Melting curve analysis was performed by bringing the temperature from 95°C to 60°C for 60 seconds at the transition rate of 1 degree per second. As Livak and Schmittgen (2001) described, for sample analysis the threshold was set based on the exponential phase of products and the 2^-ΔΔCT^ method was performed to analyze the data (19).

**Table 1:** Primer sequences used in real-time polymerase chain reaction


Gene	Primer sequencing (5´→3´)	Accession number

*ER-α*	F: TGCTTCAGGCTACCATTATGGA	NM-001122742
	R: TGGCTGGACACATATAGTCGTT	
*GdA*	F: GAGATCGTTCTGCACAGATGG	NM-001018049
	R: CGTTCGCCACCGTATAGTTGAT	
*HPRT*	F: TGGACAGGACTGAACGTCTTG	NM-000194
	R: CCAGCAGGTCAGCAAAGAATTTA	


*ER-α*; Estrogen receptor-alpha, *GdA*; Glycodelin-A, and HPRT; Hypoxanthine hosphoribosyltransferase.

## Statistical analysis

Data was analyzed by SPSS version 16 software (SPSS Inc., USA). Independent samples t test was performed to compare characteristics and hormonal profile of the fertile and the infertile women. Results are expressed as mean ± SD. Comparison of *ER-α* and *GdA* expression in studied groups was done using Mann-Whitney U-test. Spearman correlation analysis was carried out to investigate the relationship between variables. The level of significance was set at P<0.05.

## Results

**Fig.1:**
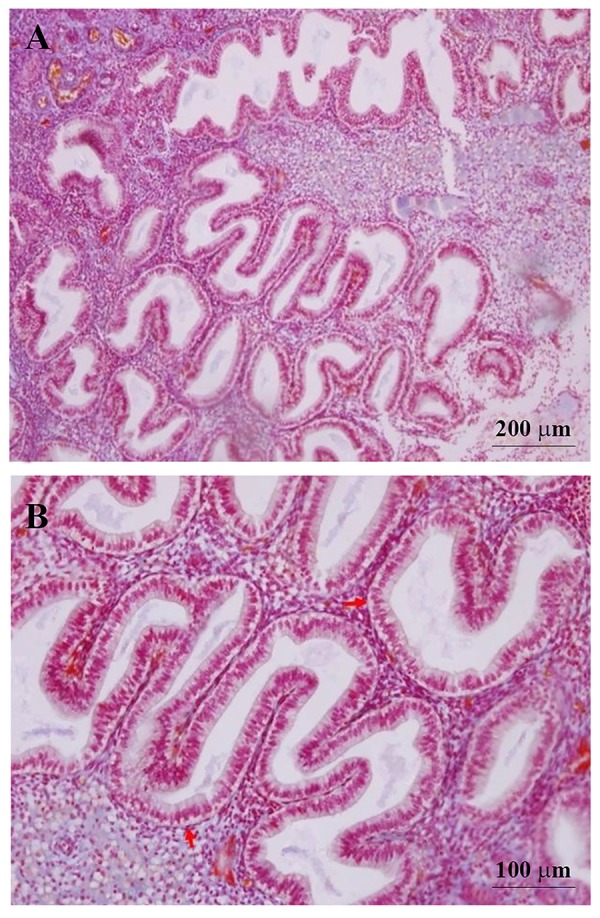
Microscopic structure of endometrium at the mid-luteal phase. A. Scale bar=200 µm and B. Scale bar=100 µm, H&E. Stromal edema and coiled endometrial glands that contain secretions with sub-nuclear vacuolization (red arrows) in their epithelium exhibit endometrium in the mid-luteal phase.

Of the 54 couples with unexplained infertility, 8 couples were excluded based on their medical records. Among 25 randomly-selected eligible patients with unexplained infertility, 9 couples refused participation. As a result, 16 infertile couples were included in the study. In addition, 10 fertile women (16.1%) out of the 62 eligible couples were included in the study. The mean age, body mass index (BMI), cycle length, duration of menses and hormonal profile in women of both groups are presented in Table 2. There were no differences in age, BMI, cycle length, duration of menses and serum LH, FSH, estradiol and progestrone concentrations between the two groups. Microscopic analysis of the endometrial biopsies showed that all samples corresponded histologically to the mid- luteal phase of endometrial cycle (Fig .1).

**Table 2:** Characteristics and hormonal profile of the fertile and infertile women in the mid-luteal phase


Parameter	Fertile women n=10	Infertile women n=16	P value

Age (Y)	31.7 ± 5.9	32.2 ± 5.5	NS
BMI (kg/m2)	23.7 ± 2.8	23.4 ± 2.6	NS
Cycle length (days)	28.2 ± 1.3	28.5 ± 1.5	NS
Menses duration (days)	4.2 ± 0.5	4.5 ± 0.6	NS
LH (mIU/mL)	12.54 ± 6.85	13.27 ± 7.13	NS
FSH (mIU/mL)	5.90 ± 2.62	6.58 ± 2.50	NS
Estradiol (pg/ml)	139.3 ± 55.4	142.9 ± 61.6	NS
Progestrone (ng/mL)	10.93 ± 3.21	11.48 ± 4.86	NS


Independent samples t test was done as the test of significant. Results expressed as mean ± SD. The level of significance was set at P<0.05. BMI; Body mass index, LH; Luteinizing hormone, FSH; Follicle stimulating hormone, and NS; Non significant.

**Fig.2:**
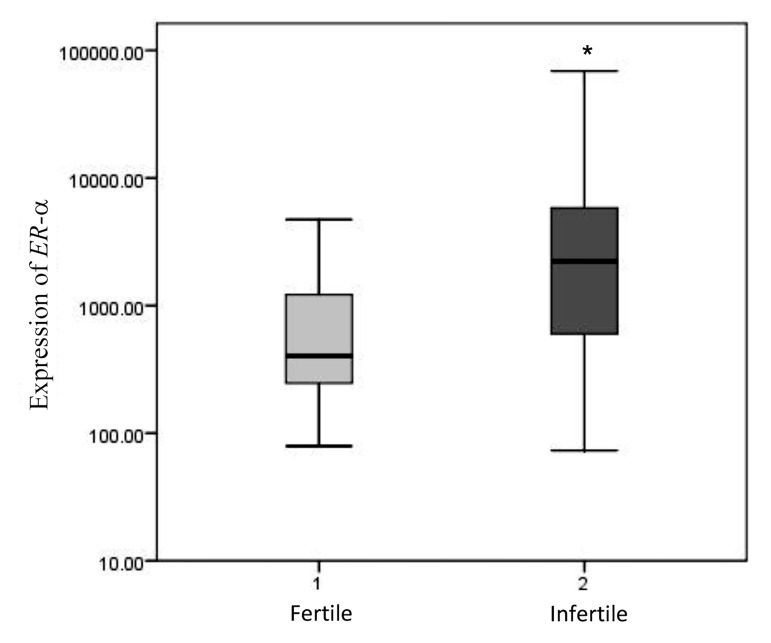
Relative expression of *ER-α* in the mid-luteal endometrium of patients with unexplained infertility (n=16) was significantly higher than those in healthy fertile women (n=10, P=0.007, Mann-Whitney U-test). *; P<0.05.

**Fig.3:**
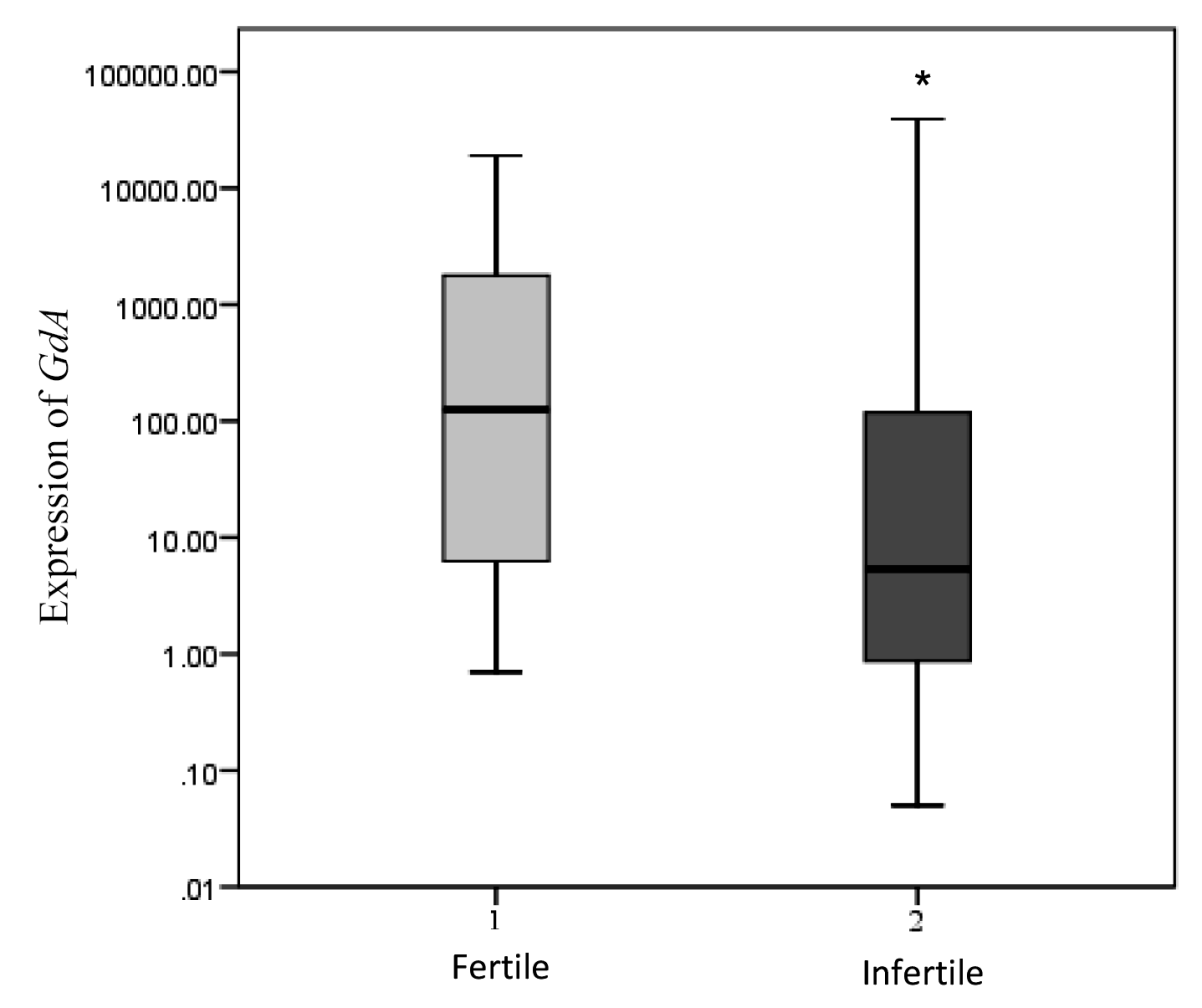
Relative expression of *GdA* in the mid-luteal endometrium of patients with unexplained infertility (n=16) was significantly lower than those in healthy fertile women (n=10, P=0.045, Mann-Whitney U-test). *; P<0.05.

Relative expressions of *ER-α* and *GdA* in the mid-luteal endometrium of the patients with unexplained infertility and healthy fertile women are shown in Figures 2 and 3. Expression levels of *ER-α* and *GdA* mRNA are given relative to the expression levels of the reference gene, HPRT. Levels of *ER-α* mRNA expression in the endometrium of the patients with unexplained infertility were significantly higher than those in the fertile women (P=0.007, Mann-Whitney U-test, Fig .2). 

*GdA* mRNA levels were significantly lower in the infertile women compared to the healthy fertile group (P=0.045, Mann-Whitney U-test, Fig .3). 

**Fig.4:**
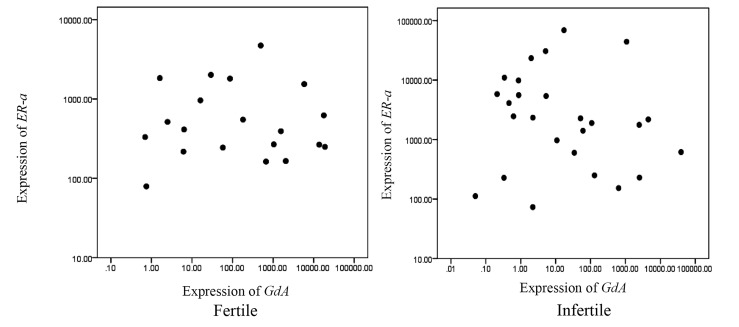
Correlation between *ER-α* and *GdA* mRNA expressions in the mid- luteal endometrium of the healthy fertile women (r=-0.047, P=0.845) and the patients with unexplained infertility (r=-0.205, P=0.316).

A statistically non-significant negative correlation was observed between *ER-α* and *GdA* mRNA expression levels in the fertile women (r=-0.047, P=0.845) and in the patients with unexplained infertility (r=-0.205, P=0.316, Fig .4). 

## Discussion

Implantation failure is believed to be a major cause of infertility (20). Successful embryo implantation depends on the development of an endometrium that is receptive to the embryo (21). Coordinated interactions between estrogen and progesterone resulting in a series of synchronized molecular events during menstrual cycle ultimately lead to the preparation of a receptive endometrium (22). 

The present study showed that a lack of appropriate levels of *ER-α* downregulation in the mid-luteal phase in the patients with unexplained infertility relative to the control group. During implantation *ER-α* is being downregulated in response to progesterone. Downregulation of *ER-α* during the mid-secretory phase is one of the primary actions of progesterone. The combination of estrogen withdrawal and progesterone action is required to stimulate the endometrial gene expression in the mid-luteal phase (8). Disappearance of *ER-α* in the mid-luteal phase provides the opportunity for progesterone to act alone specifically on the stroma (6). Paracrine activity of stroma in response to progesterone results in epithelial gene expression (7). Similar findings have been reported in patients with endometriosis and in women with PCOS (9).

Inadequate progesterone levels, defects in the progesterone receptor, hypersensitivity to estrogen, inappropriate expression of aromatase and progesterone resistance are among the reasons that can cause this failure to downregulate *ER-α* in the mid-luteal phase. Insufficient serum level of progesterone in the luteal phase defect (LPD) may delay the timing of *ER-α* downregulation during implantation (23). Resistance to progesterone due to aberrant expression or activity of receptor results in estrogenicity in endometrial tissue (24). The loss of progesterone activity caused by defect in the progesterone receptor (25) and/ or an increase in the local estrogen production due to inappropriate expression of aromatase (26) may cause the persistence of *ER-α* in endometriosis patients. A failure in *ER-α* downregulation has been reported in ovarian and peritoneal endometriosis (27). Increased production of estrogen contributes to the pathophysiology of the endometriosis as a mitogen causing aberrant proliferation (28) and inhibition of apoptosis (29). Overexpression of steroid receptor co-activators in PCOS patients which marks the hypersensitivity to estrogen may explain elevated endometrial *ER-α* expression (9).

Moreover, it seems that any change in the balance between estrogen and progesterone could disturb the timing of *ER-α* downregulation in mid-luteal phase. Endocrine disrupting chemicals (EDCs) or xenoestrogens are natural or synthetic chemicals in the diet or the environment that mimic the endogenous estrogens functions or interfere with estrogen signaling pathways (30). Lower levels of progesterone metabolite have been found during the luteal phase with higher concentration of Dichlorodiphenyldichloroethylene (DDE) (31). Impaired implantation has been reported in patients with an increase in serum 17ß-estradiol (E2) levels during the pre-implantation period, while reducing E2 levels during the pre-implantation period by a step-down protocol increases implantation and pregnancy rates (32). Accordingly, the possibility of manipulating the receptivity window with the use of different doses of E2 has been suggested (33). Aberrant uterine expression of implantation-related genes has been found at high estrogen levels (34), suggesting that in in vitro fertilization (IVF) programs estrogen levels regulation is important for improvement of women fertility.

Any inability in the *ER-α* downregulation may lead to failure to express essential proteins associated with uterine receptivity, in turn resulting in either infertility or pregnancy loss (35). The present study showes that *ER-α* overexpression is accompanied by downregulation of *GdA* in the mid-luteal endometrium of the patients with unexplained infertility. *GdA*, a potential diagnostic marker of the endometrial receptivity, is the major progesterone- regulated glycoprotein and has been demonstrated in the pinopodes of receptive-phase human endometrium (11). Lower levels of *GdA* has been reported in the secretory phase of the menstrual cycle in the eutopic tissue of patients with endometriosis (13). In addition, lower levels of *GdA* were detected in the uterine flushings on days LH+10 and LH+12 in women with unexplained infertility (14) and recurrent miscarriage (36). A negative but statistically non-significant correlation was found between *ER-α* and *GdA* in fertile women and in patients with unexplained infertility. Although transcription, synthesis, and secretion of endometrial *GdA* are regulated by progesterone, according to our findings one can assume that the overexpression of endometrial *ER-α* disturbs the expression of special genes during the implantation, which is detrimental to the development of uterine receptivity.

Inadequate uterine receptivity is responsible for approximately two-thirds of implantation failures (37). A range of cellular and molecular endometrial defects has been associated with unexplained infertility (38). Microarray analysis demonstrated that endometrial gene expression at the time of embryo implantation is considerably different in the unexplained infertile patients compared to the fertile women (39).

Therefore, the failure in *ER-α* downregulation and the observed disturbance in *GdA* expression in the patients with unexplained infertility may elucidate the causes of unexplained infertility. Our observations suggest that endometrial *ER-α* expression may participate in the cascade of molecular events leading to successful implantation.

The random inclusion of all cases diagnosed with unexplained infertility is the main strength of this study. Furthermore, real-time PCR based assay of endometrial markers, an extremely sensitive technique that allows the precise measurement of gene expression (40), increases the accuracy and external validity of our results. However, data was collected from a single randomized center and subjects represent only a fraction of the population, thus reducing the population validity. Moreover, unexplained infertile women with secondary infertility were excluded, so its external validity is restricted to women with primary infertility.

## Conclusion

The present study shows the prognostic significance of *ER-α* expression in patients with unexplained infertility. Disruption in the endometrial *ER-α* expression, which leads to defects in the uterine receptivity may contribute to unexplained infertility. In addition, our findings demonstrate that reduction in endometrial *GdA* expression was associated with elevated expression of *ER-α* in the mid-luteal phase. However, our study has some limitations including the low number of cases of unexplained infertile women with primary infertility. Studies including more tissue samples and protein-based assays such as immunohistochemistry and western blot analysis are also needed to further determine the role of endometrial *ER-α*. Understanding of biomarkers involved in the implantation and the mechanisms governing their relationships in endometrial receptivity could provide new therapeutic strategies for unexplained infertility. Whether such defects of uterine receptivity could be treated by the therapeutic blockage of *ER-α* activity or by dealing with the related causes of *ER-α* overexpression, e.g., using progestins or aromatase inhibitors to normalize the expression pattern of endometrial biomarkers associated with implantation, requires further investigation.
